# Study protocol of SWEPIS a Swedish multicentre register based randomised controlled trial to compare induction of labour at 41 completed gestational weeks versus expectant management and induction at 42 completed gestational weeks

**DOI:** 10.1186/s12884-016-0836-9

**Published:** 2016-03-07

**Authors:** Helen Elden, Henrik Hagberg, Anna Wessberg, Verena Sengpiel, Andreas Herbst, Maria Bullarbo, Christina Bergh, Kristian Bolin, Snezana Malbasic, Sissel Saltvedt, Olof Stephansson, Anna-Karin Wikström, Lars Ladfors, Ulla-Britt Wennerholm

**Affiliations:** Gothenburg University, Institute of Health and Caring Sciences, Sahlgrenska Academy, S-405 30 Gothenburg, Sweden; Gothenburg University, Perinatal centre, Department of Obstetrics and Gynecology, Institute of Clinical Sciences, Sahlgrenska Academy, Sahlgrenska University Hospital, East, S-416 85 Gothenburg, Sweden; Skane University Hospital, S-214 28 Malmo, Sweden; Gothenburg University, Reproductive Medicine, Department of Obstetrics and Gynecology, Institute of Clinical Sciences, Sahlgrenska Academy, Sahlgrenska University Hospital, S-416 85 Gothenburg, Sweden; Gothenburg University, Department of Economics and Statistics, School Business, Economics and Law, P.O. Box 640, S-405 30 Gothenburg, Sweden; South Alvsborg County Hospital, Department of Obstetrics and Gynecology, S- 501 82 Boras, Sweden; Department of Obstetrics and Gynecology, Karolinska University Hospital, 171 76 Stockholm, Sweden; Clinical Epidemiology Unit, Department of Medicine, Solna, Karolinska Institute, S-171 76 Stockholm, Sweden; Department of Women’s and Children’s Health, Division of Obstetrics and Gynecology, Solna, Karolinska University Hospital, S-171 76 Stockholm, Sweden; Uppsala University, Women’s and Children’s Health, Akademiska Hospital, SE-751 85 Uppsala, Sweden

**Keywords:** Prolonged pregnancy, Postterm, Labour, Induced, Expectant management, Perinatal outcome, Neonatal outcome, Maternal outcome, Maternal preferences

## Abstract

**Background:**

Observational data shows that postterm pregnancy (≥42 gestational weeks, GW) and late term pregnancy (≥41 GW), as compared to term pregnancy, is associated with an increased risk for adverse outcome for the mother and infant. Standard care in many countries is induction of labour at 42 GW. There is insufficient scientific support that induction of labour at 41 GW, as compared with expectant management and induction at 42 GW will reduce perinatal mortality and morbidity without an increase in operative deliveries, negative delivery experiences or higher costs. Large randomised studies are needed since important outcomes; such as perinatal mortality and hypoxic ischaemic encephalopathy are rare events.

**Methods/Design:**

A total of 10 038 healthy women ≥18 years old with a normal live singleton pregnancy in cephalic presentation at 41 GW estimated with a first or second trimester ultrasound, who is able to understand oral and written information will be randomised to labour induction at 41 GW (early induction) or expectant management and induction at 42 GW (late induction). Women will be recruited at university clinics and county hospitals in Sweden comprising more than 65 000 deliveries per year. Primary outcome will be a composite of stillbirth, neonatal mortality and severe neonatal morbidity. Secondary outcomes will be other adverse neonatal and maternal outcomes, mode of delivery, women’s experience, cost effectiveness and infant morbidity up to 3 months of age. Data on background variables, obstetric and neonatal outcomes will be obtained from the Swedish Pregnancy Register and the Swedish Neonatal Quality Register. Data on women’s experiences will be collected by questionnaires after randomisation and 3 months after delivery. Primary analysis will be intention to treat. The statistician will be blinded to group and intervention.

**Discussion:**

It is important to investigate if an intervention at 41 GW is superior to standard care in order to reduce death and lifelong disability for the children. The pregnant population, >41 GW, constitutes 15–20 % of all pregnancies and the results of the study will thus have a great impact. The use of registries for randomisation and collection of outcome data represents a unique and new study design.

**Trial registration:**

The study was registered in Current Controlled Trials, ISRCTN26113652 the 30^th^ of March 2015 (DOI 10.1186/ISRCTN26113652).

## Background

According to World Health Organisation (WHO), postterm birth is defined as pregnancy duration of 294 days or longer, i.e. gestational week (GW) 42 and 0 days (42^+0^) or more, measured from the first day of the last menstrual period [1]. This definition is arbitrary. Many studies that have analysed risks and management of postterm birth also include pregnancies from 41^+0^ GW (late term). Globally, the prevalence of postterm birth is about 5–10 % [2] but the rate varies considerably between and within countries. Factors that influence the prevalence are characteristics of the population e.g. maternal age and number of primiparous women in the population, the rate of preterm birth, interventions such as caesarean section and induction of labour, the prevalence of routine ultrasound dating of pregnancy and pregnancy surveillance routines [3]. If ultrasound is performed at 40^+0^ or 41^+0^ GW, fewer women will reach 42^+0^ GW as some pregnancies will be diagnosed as risk pregnancies leading to induction (e.g. because of oligohydramniosis or small for gestational age foetuses). The prevalence of postterm birth (42^+0^ GW or more) in Sweden was 8.4 % between 1982 and 1991 [4] and 6.9 % in 2013 [5].

The aetiology of postterm birth is largely unknown [2]. Some rare, known causes of postterm birth are foetal anencephaly, foetal adrenal hypoplasia or insufficiency and placental sulphates deficiency. Risk factors for postterm birth include: primiparity, advanced maternal age, maternal obesity, heredity, previous postterm pregnancy, and a male foetus [6–10].

### Complications in postterm pregnancies

Perinatal mortality (PNM) is defined as the prevalence of stillbirth (after GW 22^+0^) and neonatal mortality within 7 days after birth. The PNM in Sweden between 2004 and 2013 was 0.3‰ in GW 39–41 and 0.7‰ after 42 GW. A Danish national register study (*n* = 125 043 pregnancies) showed that PNM increased in women with postterm pregnancies as compared to women with term pregnancies (adjusted odds ratio (aOR) 1.36; 95 % CI 1.08–1.72) [8]. A Swedish register study of deliveries between 1982 and 1991 (*n* = 914 702 pregnancies) found significantly higher rates of stillbirth in primiparous women in GW 41 (1.86‰) and GW 42 (2.26‰) as compared to GW 40 (1.23‰) [4]. However, in multiparous women the risk of stillbirth was not significantly different in GW 41 (0.50‰) and 42 (0.86‰) as compared to GW 40 (0.53‰). These figures were calculated based on the number of women delivered in each GW. A recalculation with the number of non-delivered women in each GW (foetuses at risk) as denominator, showed significantly higher incidences of stillbirth in GW 41 and 42, as compared to GW 40, in primiparous women (0.62, 1.26 and 2.27‰, respectively), as well as in multiparous women (0.73, 1.00 and 1.51‰, respectively). Risk factors for stillbirth in postterm pregnancies are maternal age and obesity [11].

The risk of perinatal complications such as meconium aspiration syndrome (MAS), umbilical cord complications, asphyxia, pneumonia, sepsis, convulsions, shoulder dystocia, traumatic injuries and peripheral nerve damage is higher in postterm deliveries than in deliveries at term (aOR 1.1–2.0) [8]. A case-control study from Australia showed a higher risk of neonatal encephalopathy in children born postterm, (GW 41: aOR 3.3 and GW 42: aOR 13.2 versus GW 39) [12]. A Swedish study (*n* = 354 children) found an increased risk for developmental delay at the age of 4–4.5 years in children born postterm compared with children born at term [13]. Recently, also increased rates of obesity and early markers of the metabolic syndrome were found in children born postterm [14, 15]. This may not be a result of prolonged pregnancy per se but rather associated with foetal and/or parental genetic factors that also are associated with delayed parturition.

Maternal complications increase from GW 40. The risk of puerperal infections, postpartum bleeding, disproportion, labour dystocia, emergency caesarean sections, and cervical lacerations was higher for postterm than for term pregnancies in a Danish register study (*n* = 125 043 pregnancies) (aOR 1.2–1.6) [8].

## Postterm pregnancy: induction of labour or expectant management

### Randomised controlled studies (RCTs)

With the increased risks that accompany a postterm pregnancy, the obvious solution would be to suggest induction of labour before the pregnancy becomes postterm. Several RCTs have compared induction with awaiting spontaneous onset of labour, with or without foetal surveillance, but most of the studies are small and of low methodological quality. The largest study so far is a multicentre RCT from Canada (*n* = 3407). The women were randomised at 41 GW to induction or to expectant management with monitoring until the start of spontaneous labour [16]. The PNM was similarly low in the two groups but the induced group had significantly fewer caesarean sections. The study has been criticised because the induction methods were different in the groups. Prostaglandins were not used in the expectant management group, and it is speculated that this may have contributed to a higher caesarean section frequency in this group. In a later Norwegian study (*n* = 500 women), women were randomised to induction at 289 days (GW 41^+2^) or monitoring and induction at 300 days (GW 42^+6^) unless spontaneous labour [17]. The primary outcome (a composite neonatal outcome) was similar in the two groups. No case of stillbirth occurred, and there was no difference in the rate of caesarean sections. However, the study was too small to assess PNM, a problem accompanied by all RCTs to date. Several systematic reviews and meta-analyses of RCTs have been published. In a Health Technology Assessment (HTA) performed at Sahlgrenska University Hospital (SU) in Gothenburg, 13 RCTs were included (*n* = 6617 women) [18]. The review showed no difference in PNM but found significantly fewer cases of MAS and a lower caesarean section rate in the induction group as compared to the expectant management group [18]. An updated HTA meta-analysis of 17 RCTs (*n* = 7223 women) was done in 2012, with a more liberal inclusion of studies (a study from 1969, abstracts and articles in Spanish were added) [19]. It showed a lower PNM in the induction group, fewer cases of MAS, and no difference in caesarean section rate. However, a large number of women need to be induced to prevent one case of perinatal death (NNT = 469). A methodological problem in individual studies and systematic reviews is that important outcomes such as PNM and hypoxic ischaemic encephalopathy (HIE) occur at a very low frequency [20–22]. Thus, very large studies are needed to address these important outcomes.

### Observational studies

A recent register study was based on Medical Birth Registry (MBR) data on all pregnancies more than 41^+2^ weeks in Sweden between 2000 and 2007 (*n* = 119 198 pregnancies) [23]. The management of postterm pregnancy in the Stockholm region was changed in 2005 from induction of labour at GW 43^+0^ to induction at no later than GW 42^+0^. Decreases in the rates of PNM (by 48 %), MAS (by 51 %) and low Apgar scores (by 31 %) were observed. Sweden was divided into three regions based on the rate of deliveries at more than 42^+2^ weeks. It was shown that foetal surveillance differs and that regions with a high rate of deliveries at >GW 42^+2^ had a higher rate of neonates with MAS and low Apgar scores.

A recent population-based retrospective cohort study from Scotland (*n* = 1 271 549 pregnancies) showed that induction of labour for non-medical reasons at GW 37, 38, 39, 40 and 41 were associated with reduced PNM, without an increased risk of caesarean section. However, more children were admitted to the neonatal ward in the induced group [24]. An older observational study [25] compared a group of uncomplicated pregnancies between 1997 and 1999 (*n* = 2176) with a group of uncomplicated pregnancies between 1999 and 2002 (*n* = 3716). Management was changed in 1999 from induction of labour at GW 42^+0^ to induction at GW 41^+0^. There was no difference in neonatal outcome including stillbirth between the two time periods, but there were more prolonged labours and caesarean sections due to failed inductions between 1999 and 2002.

### Risks of labour induction

The RCTs referred to above [16, 17] and the systematic reviews show that induction compared with expectant policy in GW 41^+0^ or later did not increase the frequency of caesarean sections or instrumental vaginal deliveries. Earlier observational studies, comparing induction with spontaneous start of labour at the same gestational age, showed higher rates of caesarean section after induction, particularly in women with an unripe cervix [26–28]. A retrospective cohort study attempted to mimic the clinical situation by comparing women with induction in a specific GW (GW 38, 39, 40, 41) with women being undelivered in the same GW, who were either subsequently induced or went into spontaneous labour [29]. As with earlier studies, a higher frequency of caesarean sections was found after induction, which was statistically significant for GW 38, 39 and 40. At GW 41^+0^ there was no difference. A recently published meta-analysis of 31 RCTs (19 studies addressing induction for postterm birth and 12 inductions for other indications) found that induction led to a lower risk of caesarean section (OR 0.83; 95 % CI 0.76–0.92) [30]. Similar findings are reported in another recent meta-analysis of 157 RCTs [29, 31].

A register study with data from the Norwegian MBR between1996 and 1998 (*n* = 176 591 pregnancies) and the Norwegian Cerebral Palsy Register (*n* = 373 children) found an independent association between induction of labour at term and bilateral cerebral palsy (CP) (OR 3.7, 95 % CI 1.8–7.5) after adjustment for maternal disease, gestational age, birth weight, prelabour rupture of the membranes [32]. However, the authors concluded that they were only able to speculate on possible mechanisms leading to CP after labor induction due to lack of detailed data. Recently, an association between autism spectrum disorders and induced or augmented childbirth has been shown [33]. Some evidence suggests an oxytocin receptor deficiency in autism and the authors hypothesized that an oxytocin infusion may alter the oxytocin balance in the newborn.

There is no consensus as to the optimal time of labour induction or the optimal way of antenatal surveillance. Recently, guidelines from the Danish Society of Obstetrics and Gynaecology (DSOG 2011) and the Norwegian Directorate of Health (2012) have been published. According to DSOG, all pregnant women should be delivered before GW 42^+0^. A clinical examination is recommended in GW 41^+0^ including cardiotocography (CTG) and ultrasound. Induction will be planned for at GW 41^+2^–41^+5^, depending on local tradition. In Norway, a check is also recommended in GW 41^+0^–41^+2^ with a clinical examination, ultrasound, CTG and amniotomy if possible. Induction should be started no later than GW 42^+0^. Induction in GW 41 is recommended for women who are older (>38 years) or obese, who have gestational diabetes or are suspected to have a foetus being small for gestational age (SGA). The American College of Obstetricians and Gynaecologists (ACOG) recommends starting monitoring between GW 41^+0^ and 42^+0^ with CTG and measurement of the amniotic fluid index (AFI) twice a week. In Great Britain, The Royal College of Obstetricians and Gynaecologists (RCOG) recommend induction between GW 41^+0^ and 42^+0^. The Society of Obstetricians and Gynaecologists of Canada (SCOG) recommends antenatal examination in GW 41–42, with local guidelines for further action.

### Health economic considerations

The Canadian Multicentre Postterm pregnancy trial from 1995 indicated that induction of labour was associated with a lower cost as compared with expectant management of postterm pregnancy [34]. However, as the study was performed more than 20 years ago the data may not be applicable today. Induction of labor at 41 GW versus expectant management with antenatal testing in nulliparous women resulted in improved obstetric outcomes and was cost-effective when analysed with a decision-analytic model [35]. The incremental cost was $10,945 per quality-adjusted life year (QUALY) gained (the range of cost-effectiveness thresholds is commonly set at $ 50,000–100,000 per QUALY in the USA). Besides the analyses being based on a theoretical cohort, the authors stated some limitations in the cost-effective analysis. Much of the data utilized was more than 10 years old and health care costs have rapidly increased. In addition, they conducted sensitivity analysis over wide ranges of cost inputs and concluded that better cost data in this area would facilitate more accurate estimates of the cost-effectiveness of induction of labour [35].

### Women’s experiences

A Swedish study (*n* = 1111 women) found that induction of labour irrespective of cause for induction was associated with a more negative experience of childbirth [36]. This finding is inconsistent with a cross-sectional study (*n* = 252 women) from Nigeria, which showed that 71 % of the women expressed satisfaction with the induction process [37]. Only one RCT has assessed the woman’s experiences of induction or expectant policy for postterm pregnancy (*n* = 508 women) [38]. At the time of randomisation, 74 % would have preferred induction if they had been able to choose. The majority of women (84 %) who were induced had a positive childbirth experience. Despite that induction lead to more intensive labour, 74 % preferred being induced compared with awaiting spontaneous on-set of labour (*p* = 0.001).

## Aim

The aim with this study is to evaluate if a policy of induction of labour at 41 GW (early induction) is superior, in terms of neonatal and maternal outcomes, as compared to expectant management and induction at 42^+0^ (late induction) in healthy women with a low risk singleton pregnancy.

## Methods/Design

This multicentre registry based randomised controlled trial named SWEPIS (SWEdish Post-term Induction Study), will be performed at 4 university clinics and 1 county hospital in Sweden from 01-09-2015 to 31-08-2018. The centres comprise more than 65 000 deliveries per year. Eligible participants are healthy women ≥18 years old, who are able to understand oral and written information, with a normal live singleton pregnancy in cephalic presentation at 41^+0^ GW, estimated with a first or second trimester ultrasound. Women will be excluded if they have had a previous caesarean section or other uterine surgery, pregestational and insulin dependent gestational diabetes, hypertensive disorders in pregnancy including preeclampsia, multiple pregnancy, foetus in breech or transverse position, oligohydramniosis (amniotic fluid index <50 mm or deepest vertical pocket <20 mm), small for gestational age (<−22 % according to a Swedish reference [39], foetal malformations and contraindications to vaginal delivery such as placenta previa.

The Regional Ethics Board in Gothenburg approved the study in May 2014 (Dnr: 285–14).

General information of the study will be available at each antenatal care unit where women who most likely chose to give birth at any of the including hospitals will attend. All pregnant women are given oral and written information about the study at around GW 40 at the antenatal care units. All potentially eligible women will be consecutively contacted for further information. Women who are interested in participating in the study are booked for a visit at the antenatal clinic at the respective hospital at GW 41^+0^–41^+1^. Further information is given and written informed consent is obtained. Thereafter, an ultrasound examination is performed. Women with a foetus with an estimated foetal weight <22 % according to the Swedish gestational and gender adjusted reference curve and/or AFI <50 mm or DVP <20 mm will not be randomised but will be followed-up according to clinical routine. Consenting eligible women will be enrolled and randomised. Eligible women who do not want to participate in the study will be asked to fill in a web based or postal written consent for collection of information on demographic background variables, previous postterm pregnancy and obstetric and neonatal outcomes (this is to make it possible to check for selection bias).

### Randomisation

**Fig. 1:**
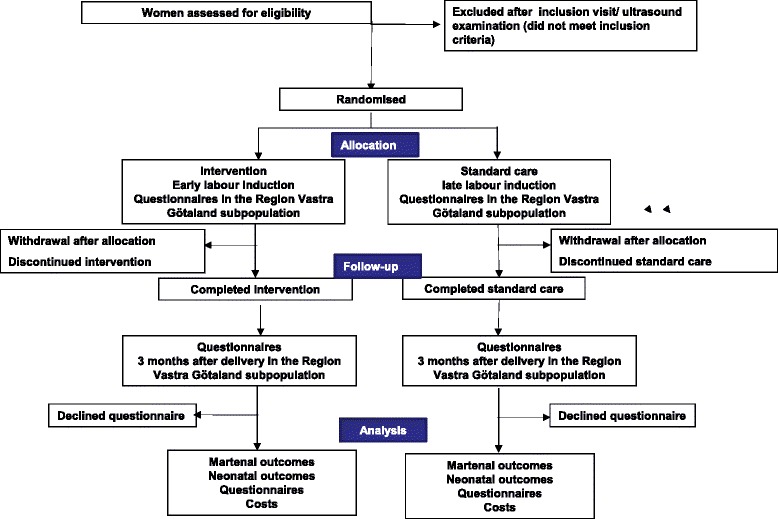
Flow-shart of the study

Figure 1 shows randomisation and progress through the trial. The doctor or midwife who performs the ultrasound will enrol eligible women. Randomisation will be completed at the antenatal clinic at GW 41^+0^ to 41^+1^. Block randomisation with a fix block size (unknown to the investigator) will be done online, 1:1, using the Pregnancy Register with a module specifically developed for the study by MedSciNet AB, Stockholm, Sweden. Stratification will be performed for centre and parity (primiparity and multiparity).

### Labour induction

Women in the intervention group (early induction) will be induced within 24 h after randomisation. Women in the control (expectant management/late induction) will be induced at GW 42^+0^ (and no later than GW 42^+1^). After randomisation, women in the control group are followed-up at the ordinary antenatal clinic according to clinical routine for GW 41^+^–42^+0^.

### Methods of labour induction (early at GW 41^+0^–41^+2^ or late at GW 42^+0^–42^+1^)

Before labour induction the following examinations are performed: blood pressure, urine analysis, abdominal and cervical examination and CTG. If there are no contraindications for induction of labour, induction will be performed according to the following:

1. if the foetal head is well engaged and the cervix is ripe (Bishop score ≥6 for primiparous and ≥5 for multiparous) amniotomy is performed. Oxytocin infusion will be started after 2 h if no or not regular contractions.
2. if the cervix is unripe (Bishop score <6) or the fetal head is not engaged any of the following methods are used (according to the routine at the clinic):
  - mechanical dilation using a Foley like catheter (BARD or Cook)
  - misoprostol (Cytotec®) orally
  - misoprostol (Misodel®) controlled-released vaginal insert
  - prostaglandin E2 (Minprostin®) gel vaginally
  - prostaglandin E2 (Propess®) vaginal insert

Mechanical dilation may be followed by prostaglandins and vice versa. If the woman is not in active labour or cervix is still unripe after 48 h, the clinician together with the woman will decide whether to perform a caesarean section or to continue expectant management until GW 42^+0^. All inductions will be performed with the patient staying at the hospital until delivery. The clinic’s protocol and guidelines for induction and surveillance during induction will be followed. During the active phase of labour fetal surveillance with continuous CTG with or without ST segment analysis (STAN) in combination with foetal scalp blood sampling is recommended.

## Outcome measures

### Primary outcome measure

A composite of stillbirth, neonatal mortality and neonatal morbidity. Stillbirth is defined as intrauterine foetal death of a foetus that was alive at randomisation. Neonatal mortality is defined as live births with death day 0–27. Neonatal morbidity is defined as at least one of the following variables: Apgar score <7 at 5 min, metabolic acidosis defined as pH <7.05 and base deficit >12 mmol/l in umbilical artery or pH <7.00 in umbilical artery, HIE I-III, intracranial haemorrhage, neonatal convulsions, meconium aspiration syndrome (MAS), mechanical ventilation, obstetric brachial plexus injury.

### Secondary outcome measures- neonates

Admittance to neonatal intensive care unit, birth weight, macrosomia (>4.5 kg), Apgar score <4 at 5 min, therapeutic hypothermia, neonatal jaundice, pneumonia, sepsis, costs.

### Secondary outcome measures-mothers

Use of epidural analgesia, caesarean section, assisted vaginal delivery, duration of labour, episiotomy, perineal lacerations III-IV, shoulder dystocia, postpartum hemorrhage (>1000 ml), chorioamnionitis, wound infection, urinary tract infection, endometritis, sepsis, costs.

Data on women’s attitudes, experiences and health-related quality of life will be collected, only in the Region Vastra Götaland subpopulation, by questionnaires (web based or postal).

#### After randomisation

Personality (Big Five), Depression/anxiety (HADS), Health related quality of life (Euro-Qol –VAS & Euro-Qol-5D, Self-efficacy (The General Self-Efficacy Scale (GES) [40–44].

Health-related quality of life (Euro-Qol –VAS & Euro-Qol-5D, the General Self-Efficacy Scale (GES),

#### Three months after delivery

Depression/anxiety (Hospital anxiety and depression scale, HADS) [44], The Childbirth Experience Questionnaire (CEQ) [41]. The instruments will be eligible in English, Spanish, Arabic, Bosnian, Croatian, Serbian, Farsi and Somali language.

For centres with the same medical record system e.g.Siemens Obstetrix (Siemens Medical solutions) we will register data on satisfaction with delivery (Visual analog scale 0–10) [45].

### Data collection

Data on background variables, obstetric and neonatal outcomes will be obtained from the Swedish Pregnancy Register and Swedish Neonatal Quality Register (SNQ) after delivery. The Pregnancy Registry is a certified national quality registry initiated by the Swedish Health care that collect and process information all the way from early pregnancy to a few months after birth. SNQ is a certified national quality registry initiated by the Swedish Health care that collect and process information about the neonate (www.snq.se).

### Statistical analysis

We anticipate that induction of labour at GW 41 as compared to induction at GW 42 will reduce the primary outcome by one third from 2.74 to 1.84 % (level of significance 0.05, power 80 %, dropout rate 10 %). The primary composite outcome of 2.74 % is based on the following rates of perinatal mortality and neonatal morbidity in GW 41^+3^ from Region Skane between 2000 and 2010: HIE 0.22 %, MAS 0.36 %, obstetric brachial plexus injury 0.05 %, Apgar score <7 at 5 min 1.58 %, stillbirth and neonatal mortality 0.53 %. We need a sample size of 10 038 women to be randomised, 5 019 to induction of labour at GW 41^+0^ and 5 019 to expectant management. Since primary outcome is a serious and rare event, we consider that a 33 % reduction is clinically relevant. In 2013, Region of Vastra Gotaland, Region Skane, Stockholm and Uppsala had 18 261, 14 719, 28 335 and 3 787 deliveries, respectively, altogether 65 102 deliveries. During a 3 year period we calculate with 195 306 deliveries. Given a rate of 18 % of women being undelivered at 41^+0^ GW, this will give us 34 755 women during 3 years. We estimate that around 33 % (11 469) of these women can be included.

The primary statistical analysis will be the comparison of primary composite outcome (stillbirth, neonatal mortality and morbidity) between the two randomised groups with two-sided Fisher’s exact test on the intention to treat (ITT) [46] populations at significance level 0.05. Adjustments for baseline variables if necessary and interaction analyses will be performed with multivariable logistic regression analyses. For variables with interactions *p* <0.10 with primary efficacy variable, subgroup analyses will be performed. Women with spontaneous labour or prelabour rupture of the membranes (PROM) after randomisation but before planned induction will be included in the statistical analysis according to ITT. Women with pregnancy complications after randomisation indicating any need of intervention are included in the statistical analysis according to ITT. Foetuses/newborns with lethal birth defects will be excluded in the analysis of stillbirth, neonatal mortality and morbidity. The median, 95 % CI, quartiles, means and SD are calculated when appropriate. The Mann–Whitney U test is used to compare differences between the two groups concerning continuous variables, Fisher’s exact test for dichotomous variables, Mantel-Haenszel Chi-square test for ordered categorical variables and Chi-square test for unordered categorical variables.

The statistician will be blinded to group and intervention. Otherwise blinding is not possible for practical reasons.

### Cost-effectiveness analyses

Cost-effectiveness analyses will be performed comparing induction at GW 41^+0^ with induction at GW 42^+0^. The primary measures of effectiveness will be a composite of stillbirth, neonatal mortality and neonatal morbidity (i.e. primary outcome) [47]. A broad range of sensitivity analyses will be performed, which includes calculating CIs of the cost-effectiveness measures, using bootstrapping [47]. In order to extrapolate the outcomes observed in this study to a longer time span we will utilize a simulation model. The model will be constructed and parameterized using estimates of relevant parameters obtained within the project. Moreover, associations between neonatal outcomes and health outcomes later in life that was not studied in this project will be collected from the epidemiology literature. Initially, two modelling structures will be considered: a Markov structure and a discrete event structure [48]. The typical Markov model uses a cohort and state-transition technique, while the discrete event structure means that simulations are event-based and performed at the individual level. The choice between these two main alternatives will be performed according to the guidelines provided in Brennan et al. [48].

### Serious adverse events

The following serious adverse events (SAE) will be identified: stillbirth, neonatal death, severe neonatal morbidity, defined as intraventricular haemorrhage, asphyxia or MAS with admission to NICU, maternal death, severe maternal morbidity defined as admission to intensive care unit and events related to induction of labour e.g. uterine rupture. All SAEs are reported to the main investigators, who will report to the Data and Safety Monitoring Board (DSMB).

### Data safety

The steering committee’ is responsible for the study design, the co-ordination between centres, the progress of the study, and for the results being statistically analysed and summarised for publication. The steering committee are also principal investigators, i.e. they are responsible for the project at their own centre for example adherence to the study protocol, recruitment and any practical problem that may arise within the frame of the project. The DSMB consist of experts (one statistician, a senior obstetrician and a midwife) who will supervise the study. The DSMB will secure the project management through periodical reviews and will make the recommendations concerning the continuation, modification or termination of the trial.

## Trial status

Besides the literature search resulting in the systematic review and meta-analysis and the HTA reports [18, 19] there are no clinical results. Overall recruitment start date: 01/09/2015 and overall trial end date: 31/12/2018.

## Discussion

The planned study is the first RCT utilising the national Pregnancy Register (developed by co-applicant OS) for collecting data on maternal characteristics, pregnancy, delivery and postnatal care and SNQ for data on neonatal outcome. Randomisation will be performed using a computerised module linked to the Pregnancy Register. Such a design has recently been applied successfully in cardiovascular research [49, 50] and has several important advantages including costs, ease of recruitment and facilitating the translation of the study results to clinical practice.

Our primary and secondary outcome measures are in the interest of the woman, the expectant partner and the child. There are no high quality studies with adequate sample size investigating if early induction is superior to standard care (expectant management/late induction) in terms of maternal and neonatal outcomes. The optimal timing of offering induction of labour to women at late term or postterm therefore needs further investigation. Women’s experiences and opinions about the choices have not been adequately evaluated. In this study, we also aim to evaluate women’s attitudes and experiences with questionnaires. In the early induction group we will have a higher proportion of induction of labour, with possible negative delivery experiences. In the late induction group, the proportion of induction will be lower, but the experience of another week of waiting “post due date” is not evaluated. Further, the foetal growth continues between 41 and 42 GW, leading to an expected higher infant birth weight and more “macrosomic infants” (>4.5 kg) in the late compared to the early induction group. Giving birth to macrosomic infants is associated with more complications, leading to a possible more negative experience in the late induction group. The outcome “women’s experiences and opinions” of each intervention (early or late induction) is thus complex and therefore an important outcome of our study. One of the advantages of the register-based design is that the primary and secondary outcomes will be collected continuously through national quality and health registers. These data can be used for implementation after the study has been finalized. The consequences of a new clinical management can be evaluated (reduction in e.g. perinatal mortality, HIE, caesarean section etc.) using national data from the Pregnancy Register and SNQ. The study results will fill several gaps in health care knowledge and contribute to establish good evidence-based routines regarding management of postterm pregnancy. Support for if an early intervention in the natural course of pregnancy is superior for the health of children and mothers is important, to justify an intervention (labour induction) in 15–20 % of all women whose pregnancy has lasted ≥41^+0^ GW in the future. Sweden is suited to run a high quality RCT: public health care system, almost 100 % of pregnant women attend antenatal care, nearly everybody gives birth at a delivery hospital and there is usually (except the Stockholm region) only one hospital with a delivery ward for a certain geographic region (reduces selection bias), low rates of maternal and perinatal mortality, comparably low caesarean section rates and opposed to many other countries we have not yet started to induce earlier so we still have the possibility to run this RCT. Also, this project is the first study supported by the recently started Swedish National Network on Clinical Studies in obstetrics and gynaecology (SNAKS). The network comprises one member from each university region in Sweden and is supported by the Swedish Society of Obstetrics and Gynaecology (SFOG). If the intervention, early induction, is shown to be superior to expectant management/late induction, new local, regional and national guidelines may therefore be developed within a short time frame (approximately 3 years).

### Project organization and co-workers

The following five centres will participate in the study: the Departments of Obstetrics and Gynecology at South Alvsborg County Hospital (SAS), Sahlgrenska University Hospital, Skane University Hospital (SUS), Karolinska Hospital Solna/Huddinge, Stockholm, Uppsala University Hospital. Our aim is to include most of the centres in Sweden through SNAKS. The project will be led by a steering committee which preliminarily has the following members: Associate professor Ulla-Britt Wennerholm, Professor Henrik Hagberg, Professor Christina Bergh, Associate professor Andreas Herbst, PhD Maria Bullarbo, PhD Helen Elden, PhD Verena Sengpiel, PhD Sissel Saltvedt, Associate professor Olof Stephansson, Associate professor Anna-Karin Wikström and Associate professor Lars Ladfors. The steering committee is responsible for the study design, the co-ordination between centres, the progress of the study, and for the results being analysed and summarised for publication. The steering committee are also principal investigators, i.e. they are responsible for the project at their own centre for example adherence to the study protocol, recruitment and any practical problem that may arise within the frame of the project.

There will be further coordinating clinical investigators at each site. One midwife will be responsible for the study implementation at each site. At SU this will be Anna Wessberg, midwife and registered PhD student. Professor in Health Economics Kristian Bolin is responsible for the health economic analysis and Nils Gunnar Pehrsson is responsible for the statistical analysis.

## Abbreviations

ACOG: American College of Obstetricians and Gynaecologists
AFI: amniotic fluid index
aOR: adjusted odds ratio
BMI: body mass Index
CI: confidence interval
CTG: cardiotocography
DSMB: data and safety monitoring board
DSOG: Danish Society of Obstetrics and Gynaecology
eCRF: electronic case record form
GW: gestational week
HADS: hospital anxiety and depression scale
HIE: ischaemic encephalopathy
HTA: health technology assessment
ITT: intention to treat
MAS: meconium aspiration syndrome
MBR: medical birth registry
NNT: numbers needed to treat
NM: neonatal mortality
PNM: perinatal mortality
QUALYS: quality-adjusted life year
RCT: randomised controlled trial
RCOG: Royal College of Obstetricians and Gynaecologists
SAE: serious adverse events
SAS: South Alvsborg County Hospital
SCOG: Society of Obstetricians and Gynaecologists of Canada
SD: standard deviation
SFOG: Swedish Society of Obstetrics and Gynaecology
SNAKS: Swedish Network on Clinical Studies
SNQ: Swedish Neonatal Quality Register
SU: Sahlgrenska University Hospital
SUS: Skane University Hospital
SWEPIS: SWEdish Post-term Induction Study
VAS: visual analog scale
WHO: World Health Organisation
